# Improvement of microwave absorption properties of polyester coatings using NiFe_2_O_4_, X-doped g-C_3_N_4_ (X = S, P, and O), and MTiO_3_ (M = Fe, Mg, and Zn) nanofillers

**DOI:** 10.1038/s41598-021-98666-6

**Published:** 2021-09-29

**Authors:** Somayeh Solgi, Mir Saeed Seyed Dorraji, Seyyedeh Fatemeh Hosseini, Mohammad Hossein Rasoulifard, Ismael Hajimiri, Alireza Amani-Ghadim

**Affiliations:** 1 Applied Chemistry Research Laboratory, Department of Chemistry, Faculty of Science, University of Zanjan, Zanjan, Iran; 2 Department of Chemistry, Faculty of Science, Azarbaijan Shahid Madani University, P.O. Box 83714-161, Tabriz, Iran

**Keywords:** Electrical and electronic engineering, Condensed-matter physics, Materials chemistry

## Abstract

In recent decades, to reduce electromagnetic pollution, scientists focus on finding new microwave absorbers with effective performance, thin thickness, and broad bandwidth. In this work, the nanoparticles of NiFe_2_O_4_, X-doped g-C_3_N_4_ (M = S, P, and O), and MTiO_3_ (M = Fe, Mg, and Zn) were successfully synthesized using co-precipitation, specific heat program, and semi-wet sol–gel methods, respectively. The synthesized nanoparticles were utilized as absorption agents and polyester resin as the matrix. Morphology, particle size, crystal structure, and chemical composition of the prepared nanocomposites were characterized by scanning electron microscope (SEM), transmission electron microscope (TEM), X-ray diffractometer (XRD), and energy dispersive X-Ray analysis (EDX), respectively. The microwave absorption performance of the coatings was also investigated by a vector network analyzer (VNA). Moreover, the effect of different parameters on the performance of absorbent coatings was studied by the Taguchi method and optimized to achieve an optimal absorbent. The results showed that the optimal nanocomposite has the reflectance loss (RL) less than − 30 dB (equal to absorption > 99%) at a high-frequency range (8–12 GHz) and 1 mm thickness. Furthermore, the addition of such novel nanoparticles to absorbents resulted in high values of attenuation constant (more than 200 dB/m) at the X-band. Therefore, the polyester coating filled with ZnTiO_3_, O-doped g-C_3_N_4_, and NiFe_2_O_4_ nanofillers can be considered a high-efficiency and low-density absorber.

## Introduction

Nowadays, electromagnetic pollution (PE) generated by telecommunication and electrical facilities has become a serious problem in people's daily life. Excessive radiation of electromagnetic waves causes adverse effects on human health, the environment, and plant growth. Therefore, the high-performance microwave absorbers have attracted the attention of many researchers in the field of decreasing unwanted emissions of electromagnetic waves^1–4^. The microwave absorbing materials are divided into: dielectric and magnetic, depending on the reaction mechanism between the absorber and the wave^5^. Besides, according to the theory of electromagnetic energy transfer, there are two basic requirements for effective microwave absorption: (1) Appropriate impedance matching should be considered between the magnetic loss and the dielectric loss; (2) The wave must be attenuated to a large extent after the incident with the material and transferring from within it. The scientists are still looking for a way to meet both requirements at the same time. According to previous reports, using one material as a microwave absorber cannot meet these two requirements. Meanwhile, the combination of dielectric and magnetic materials can provide a promising solution to improve the performance of the microwave absorbers^6–9^.

In recent years, spinel ferrites have been widely studied as microwave absorbent materials due to high magnetic permeability, spinel structure, low eddy current losses, and excellent magnetic features. Generally, the spinel ferrites are known by the general formula MFe_2_O_4_, where M is the transition metal such as nickel, zinc, magnesium, cobalt, calcium, and manganese. The spinel structure has a space group of R_3_M^10–13^. However, the ferrites have a high density; it can be improved by reducing the particle size, which not only decreases the specific gravity but also increases the microwave absorption properties. In particular, the nanoparticles of nickel ferrite (NiFe_2_O_4_) with the soft spinel ferrite structure have attracted the attention of many researchers owing to their excellent chemical stability, good thermal stability, modest saturation magnetization, availability, lightweight, and high electrical resistivity. These nanoparticles have also been employed as catalysts, pigment, MRI contrast agents, biomedical agents in drug delivery, photo-magnetic materials, sensors, etc.^8,14,15^.

Scientists today have conducted in-depth studies to find materials with high dielectric constant^16^. They can be classified as capacitor materials with high dielectric permittivity such as ceramics^17^ and microwave dielectric materials with low permittivity such as graphite carbon nitrite^18^, reduced graphene oxide^19,20^, carbon nanotube^21,22^. In the present work, MTiO_3_ (M = Fe, Mg, and Zn) nanoparticles were applied as dielectric materials with high permittivity. These dielectric materials are titanium-based ceramic oxides that have a space group of R_3_M. These materials have received much attention in the field of microwave wave absorption due to their outstanding properties such as low density, high thermal stability, and high dielectric constant^23–25^. On the other hand, conductor fillers, microwave dielectric materials, are widely used in the absorbers to lose electromagnetic energy, such as conductive polymers, graphite, CNT, etc. Among these materials, the graphitic carbon nitride (g-C_3_N_4_) has attracted the attention of many researchers as a nonmetallic conductive material due to its unique properties such as high dielectric constant, excellent conductivity, and high specific area. Besides, the g-C_3_N_4_ conductivity increases by inserting phosphorus, oxygen, and sulfur atoms into the g-C_3_N_4_ lattice structure instead of carbon atoms, which may be due to rising electron motion^16,26,27^.

In this work, the nanofillers of NiFe_2_O_4_, X-doped g-C_3_N_4_ (M = S, P, and O), and MTiO_3_ (M = Fe, Mg, and Zn) were successfully synthesized as the magnetic, conductive, and dielectric materials, respectively. Then, they were inserted into the polyester resin as a substrate. The structure and microwave absorption performance of the synthesized samples were carefully investigated. Moreover, the purpose of this work was to study the impact of different parameters on the performance of prepared absorbers in terms of improving microwave absorption and impedance matching in the X-band while keeping the thickness to a minimum. These parameters include the type of dielectric material, the type of dopant in g-C_3_N_4_, the weight ratio of MTiO_3_:NiFe_2_O_4_, and the weight ratio of MTiO_3_/NiFe_2_O_4_: X-doped g-C_3_N_4_. According to the results obtained, the synthesized nanocomposite under the optimal conditions has higher attenuation, good impendence matching, and effective absorption in X-band. Therefore, this nanocomposite containing ZnTiO_3_, O-doped g-C_3_N_4_, NiFe_2_O_4_, and polyester can be used as an enhanced microwave absorber.

## Experimental details

### Materials

Ni (NO_3_)_2_.6H_2_O (99%), FeCl_3_ (99%), NaOH (98%), TiO_2_ (99.5%), FeSO_4_.7H_2_O (98%), ZnSO_4_.7H_2_O (98%), MgSO_4_.7H_2_O (98%), thiourea (98%), melamine (99.5%), oxalic acid (99%), di-ammonium hydrogen phosphate (99%), citric acid (99.5%), and ethanol (98%) were received from Merck, Germany. Cobalt(II) naphthenate accelerator solution, peroxide hardener, *N*, *N*-dimethyl aniline, and polyester resin were purchased from Resitan Co., Iran. All chemicals were used without purification.

### Instruments and measurements

The phase structure and crystallinity of the nanofillers were investigated by a Bruker D8 Advance X-ray diffractometer (XRD) with Cu Kα radiation (λ = 1.5418 Å) in the Bragg–Brentano geometry. The scanning electron microscope (SEM, Seron Technology, Gyeonggi-do, Korea) equipped with the energy-dispersive X-ray spectroscope (EDX) was applied to determine the morphology and chemical composition of the prepared nanocomposites. In the last technique, all the samples were prepared with a coating of gold. Analysis and processing of SEM images were performed using the Image J software (version 1.53k; https://imagej.nih.gov/ij/download.html). The distribution of the nanofillers in the composite was confirmed using SEM/EDX elemental maps. Transmission electron microscope (TEM, Philips EM208S 100 kV) was used to examine the sample microstructures. A vector network analyzer (HP8410C, USA) over the frequency domain of 8–12 GHz (X-band) was utilized to measure the scattering parameters of the synthesized samples. For this purpose, the samples were first prepared under indicated conditions by the Taguchi method, and then they were cast on the rectangular molds of 10.2 mm × 22.9 mm × 1 mm.

### Synthesis of NiFe_2_O_4_ nanoparticles

The nanoparticles of NiFe_2_O_4_ were synthesized through the co-precipitation method. First, FeCl_3_ and Ni (NO_3_)_2_.6H_2_O were dissolved with a molar ratio of 2:1 in a specified amount of distilled water under stirring for half an hour. Then, the aqueous solution of NaOH was slowly added to the mixture as a precipitating agent until the pH reached 11.5. The obtained liquid-sediment was stirred using a magnetic stirrer at 80 °C for 60 min. Finally, the solid product was calcined in a furnace at 800 °C for 5 h. The powder obtained after cooling by a mortar was well ground.

### Synthesis of MTiO_3_ (M = Fe, Mg, and Zn)

The semi-wet sol–gel method was used to prepare nanoparticles of FeTiO_3_, MgTiO_3_, and ZnTiO_3_. In summary, to prepare the FeTiO_3_ nanoparticles, TiO_2_, FeSO_4_⋅7H_2_O, and citric acid were dissolved with a 2:1:1 molar ratio in distilled water. Then, the resulting solution was heated to 80 °C under stirring for water removal. Finally, the obtained sediment was calcined in a furnace at 800 °C for 8 h. After calcination, the product was well ground to get a uniform powder. Similarly, for the synthesis of MgTiO_3_, and ZnTiO_3_ nanoparticles, all of the above steps were repeated except that MgSO_4_⋅7H_2_O and ZnSO_4_⋅7H_2_O were used instead of FeSO_4_.7H_2_O in the initial stage, respectively.

### Synthesis of X-doped g-C_3_N_4_ (M = S, P, and O) nanoparticles

The nanoparticles of O-g-C_3_N_4_, P-g-C_3_N_4_, and S-g-C_3_N_4_ were prepared using a specific heat program method. According to this method, to prepare the nanoparticles of S-g-C_3_N_4_, 10 g of melamine and 6 g of oxalic acid were dissolved in 50 ml of distilled water and ethanol mixture (with a molar ratio of 1:1). Then, the resultant solution was stirred by a magnetic stirrer for 1 h, in which hydrogen interactions between oxalic acid and melamine occurred during this period. The mixture was heated to 100 °C under stirring to remove the solvent. Then, the obtained powder was dried into an oven at 120 °C and calcined into a furnace at 530 °C for 3 h. Finally, the resultant product was washed with a mixture of distilled water and ethanol (with a molar ratio of 1:1) and ground with mortar. Similarly, to the synthesis of P-g-C_3_N_4_, and S-g-C_3_N_4_, all the above steps were repeated except that di-ammonium hydrogen phosphate and thiourea were used instead of oxalic acid.

### Experimental design

The experimental design by the Taguchi method provides a significant reduction in costs, time, and the number of experiments that have been widely used by researchers in recent years. In the Taguchi method, the orthogonal arrays are applied for data analysis and experiment arrangement, depending on the number of selected factors and their levels. Moreover, the signal-to-noise ratio (S/N) is defined as the changes made by a factor to improve the quality, in which the highest S/N is chosen as the optimal conditions^28^. In this work, the L_9_ (3^4) orthogonal array was selected (Table 1) according to the number of controllable factors (4) and their variation levels (3), as shown in Table 2. Besides, the higher the better was considered as the quality index, which S/N for each output is obtained by the following equation:

1 $$\frac{S}{N} = - 10\log \left[ {\frac{1}{n}\mathop \sum \limits_{i = 1}^{n} \frac{1}{{y_{i}^{2} }}} \right]$$

**Table 1:** Parameters and their levels in the Taguchi experimental design.

Parameter	Level		
	1	2	3
The type of dielectric material	Mg	Fe	Zn
The type of dopant in g-C_3_N_4_	S	O	P
The weight ratio of MTiO_3_:NiFe_2_O_4_	1:2	1:1	2:1
The total weight ratio of MTiO_3_/NiFe_2_O_4_: X-doped g-C_3_N_4_	1:2	1:1	2:1

**Table 2:** L_9_ (3^4^) orthogonal array.

Test	Parameters			
	1	2	3	4
1	1	1	1	1
2	1	2	2	2
3	1	3	3	3
4	2	1	2	3
5	2	2	3	1
6	2	3	1	2
7	3	1	3	2
8	3	2	1	3
9	3	3	2	1

where n is the number of experiments, and Y_i_ is the experiment’s results^29^. In the present work, each test was performed three times to evaluate the repeatability of the measurements. The statistical analysis of the data was carried out by using Qualitek-4 software (version 15.6+; http://nutek-us.com/wp-dm4.html).

### Preparation of nanocomposite

**Figure 1:**
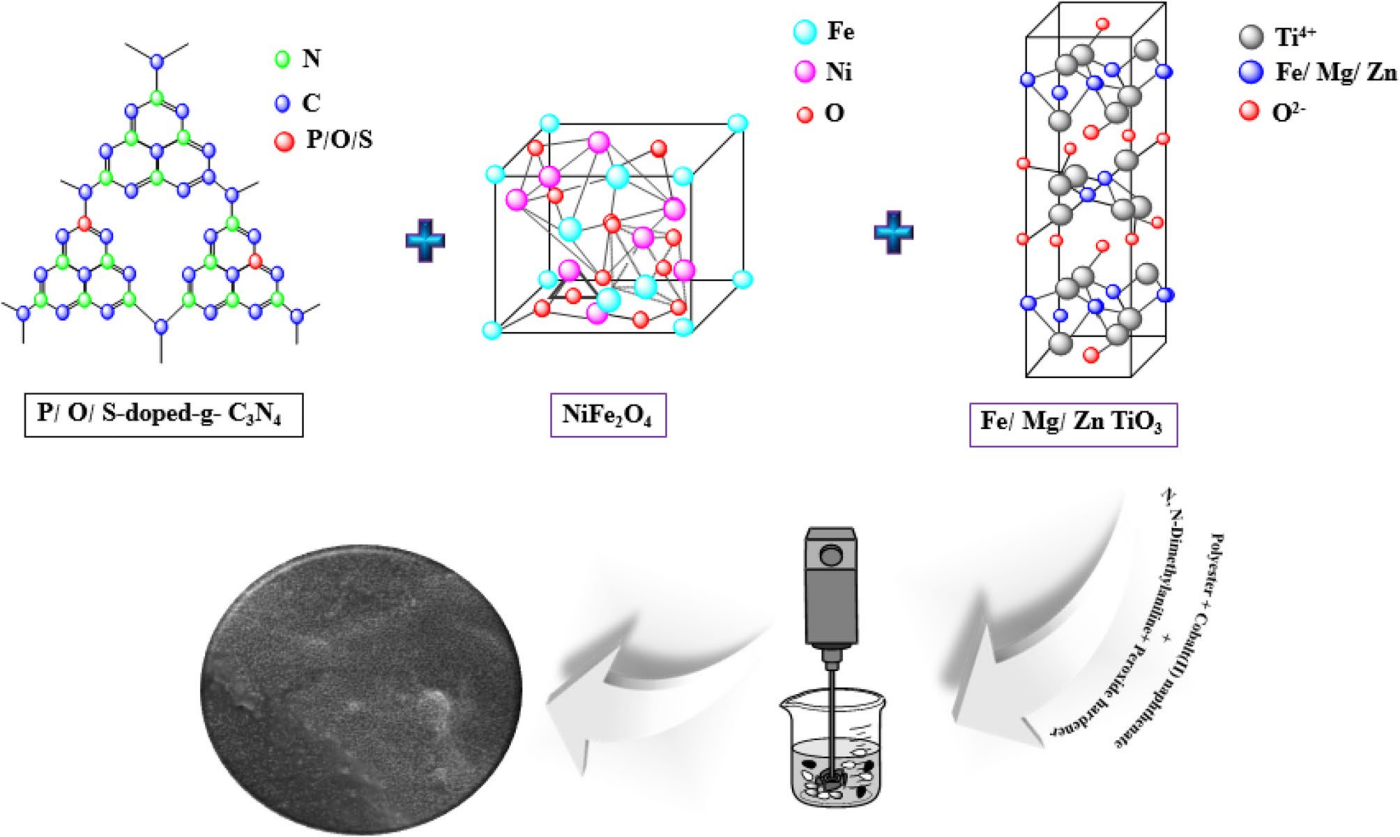
Schematic illustration of the synthesis procedure of NiFe_2_O_4_, X-doped g-C_3_N_4_ (X = S, P, and O), MTiO_3_ (M = Fe, Mg, and Zn), and polyester nanocomposite.

To prepare the absorbent coatings, the filler values in the polyester substrate were first weighed according to Table 2. 0.04 wt% N, N-dimethyl aniline, and 0.05 wt% cobalt(II) naphthenate accelerator solution were added to the polyester resin under stirring for 5 min. Then, specific amounts of fillers were added to the mixture and mixed by a mechanical stirrer until the uniform distribution was obtained. After de-oxygenation, 0.04 wt% of peroxide hardener was added to the mixture and stirred for 2 min. In the long run, the mixture was transferred to the rectangular templates of 10.2 mm × 22.9 mm × 1 mm. The synthesis procedure is schematically shown in Fig. 1.

## Results and discussion

### Characterizations

**Figure 2:**
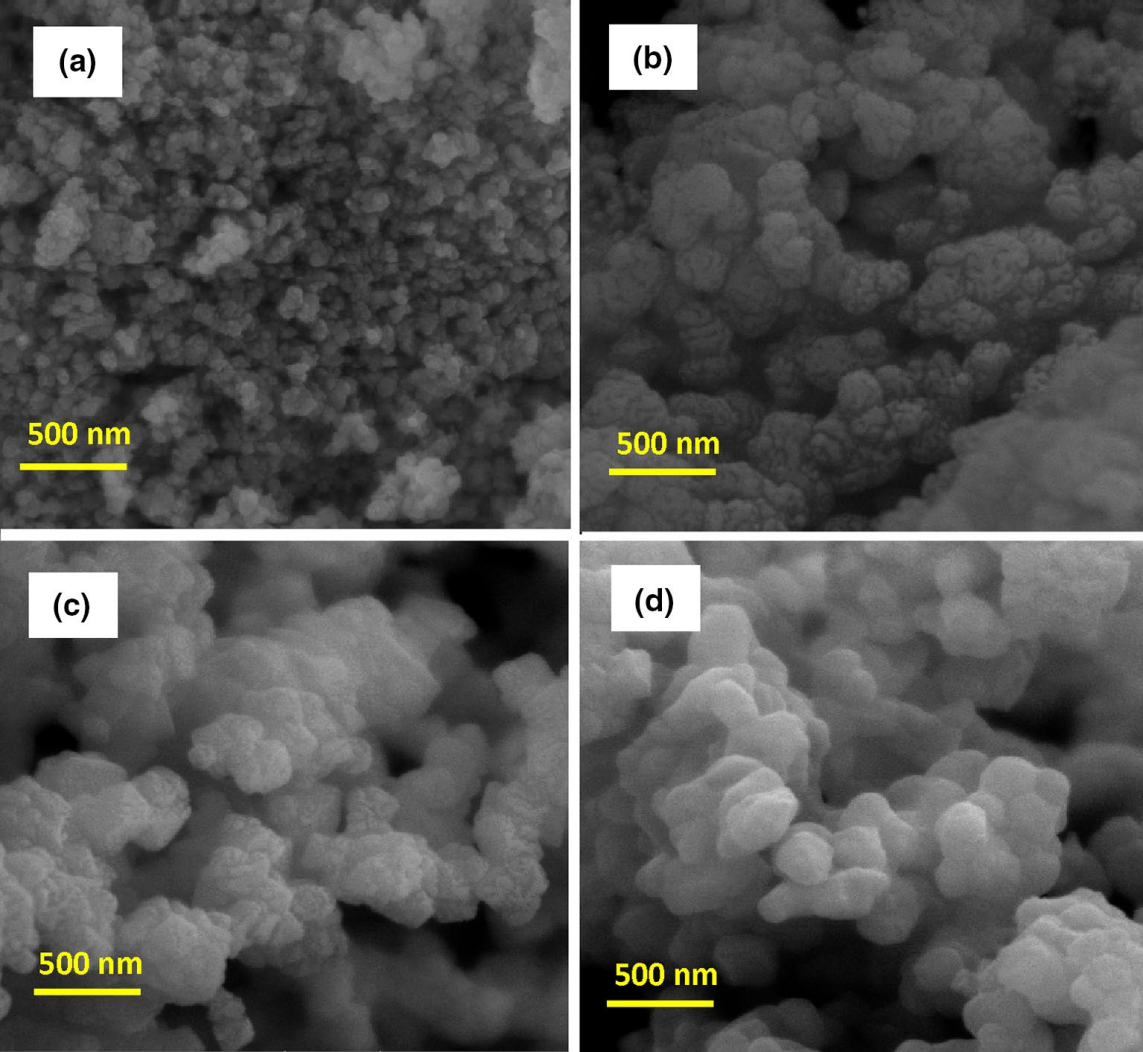
SEM images of (**a**) NiFe_2_O_4_; (**b**) MgTiO_3_; (**c**) ZnTiO_3_; (**d**) FeTiO_3_ nanoparticles.

**Figure 3:**
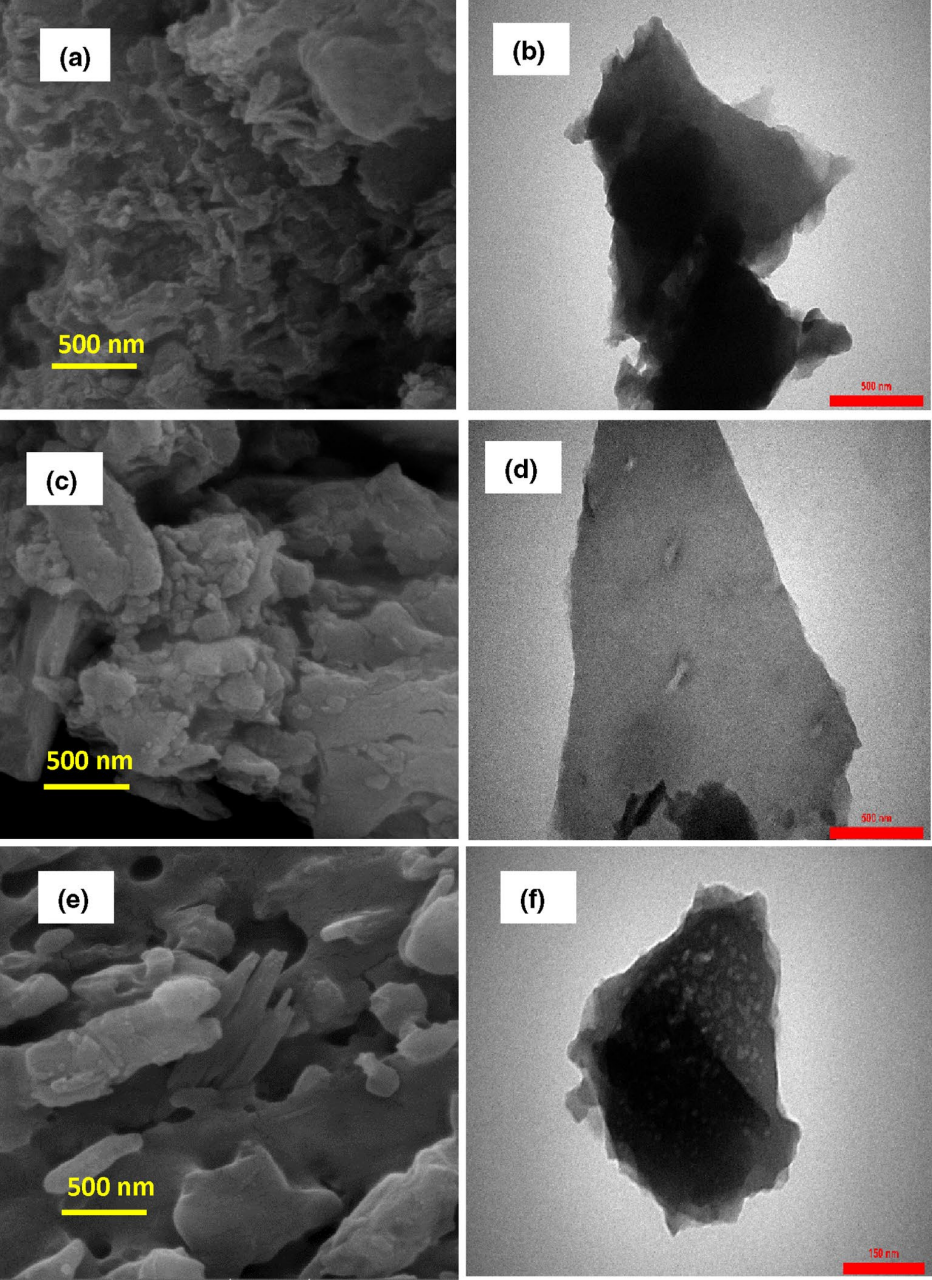
SEM images of (**a**) O-g-C_3_N_4_; (**c**) S-g-C_3_N_4_; (**e**) P-g-C_3_N_4_ nanoparticles; and TEM images of (**b**) O-g-C_3_N_4_; (**d**) S-g-C_3_N_4_; (**f**) P-g-C_3_N_4_.

The XRD pattern of the prepared NiFe_2_O_4_ nanoparticles as the magnetic filler is revealed in Fig. S1 (Supporting Information). The presence of distinct peaks in this figure indicates that the particles are well separated from each other. The diffraction peaks at the different angles of 30.3°, 35.8°, 37.3°, 89.4°, 54.4°, 57.4°, and 62.9° are attributed to Miller indices of (220), (311), (222), (400), (422), (511), and (440), respectively. As a result, this XRD pattern was well-matched with the JCPDS standard file No. 10-0325, which confirm the formation of the single phase of iron oxide with the cubic spinel structure. Moreover, the sharp peaks show that the synthesized nanoparticles have high crystallinity. However, these peaks were broadly distributed, owing to the scattering of X-rays from small particles. In addition, the peaks of NiO and α-Fe_2_O_3_ as impurity could be perceived in this pattern, which is probably due to Ni oxidation during the preparation process of NiFe_2_O_4_, which causes some Ni ions to be lost, so the presence of excess Fe ions will form α-Fe_2_O_3_ particles. Besides, the average crystallite size (L) of the prepared nanoparticles was obtained as 37.22 nm by using the Debye–Scherrer formula, as is shown below:

2 $$B \left( {2\theta } \right) = {\raise0.7ex\hbox{${K\lambda }$} \!\mathord{\left/ {\vphantom {{K\lambda } {L \cdot \cos \theta }}}\right.\kern-\nulldelimiterspace} \!\lower0.7ex\hbox{${L \cdot \cos \theta }$}}$$

where θ is the angle of diffraction, B is the peak width in a specified amount of 2θ, K is a constant that its value depends on the geometrical shape of the particles^30,31^. Figure S2a (Supporting Information) shows the X-ray diffraction pattern of the synthesized MgTiO_3_ nanoparticles. According to this pattern, the main diffraction peaks were detected at (003), (101), (012), (104), (110), (113), (024), (116), (018), (214), and (300), which was matched well with JCPDS card No. 06-0494. In addition to these sharp and narrow peaks indicate the high crystallinity and formation of the cubic structure in MgTiO_3_ nanoparticles with lattice constants of a = b = 5.05831 Å and c = 13.90858 Å and also bond angles of α = β = 90° and γ = 120°. No peaks of impurities were observed in this pattern, which can ascribe to the high purity of the synthesized MgTiO_3_ nanoparticles. The crystallite size of MgTiO_3_ is about 53.94 nm, according to the Scherrer formula. Figure S2b (Supporting Information) displays the XRD pattern of the prepared magnetic nanoparticles of ZnTiO_3_. The main peaks of ZnTiO_3_ were observed at (104), (110), (116), (018), and (214), which are in good agreement with the standard file of JCPDS card No. 00-039-0190. The strong and sharp peaks of ZnTiO_3_ confirm the formation of the cubic morphology with lattice constants of a = b = 5.078 Å and c = 9.27 Å and the high crystallinity. However, the impure peaks related to Zn_2_TiO_4_ phases were detected in the XRD pattern due to the annealing at a high temperature. The crystallite size of ZnTiO_3_ was calculated at 45.31 nm. The XRD pattern of FeTiO_3_ nanoparticles is revealed in Fig. S2c (Supporting Information). The index peaks located at (103), (015), (006), (115), (300), and (303) are in accordance with the JCPDS card No. 75-1207. The existence of strong and sharp peaks implies the high crystallinity of the synthesized nanoparticles and the formation of the cubic crystal structure with lattice constants of a = 5.141 Å and c = 14.22 Å. However, the impurity peaks ascribed to the anatase and rutile phases of TiO_2_ crystals appeared in this pattern. Besides, the crystallite size of FeTiO_3_ was obtained at about 54.80 nm. Figure S3a–c (Supporting Information) represents the X-ray diffraction patterns of O-g-C_3_N_4_, S-g-C_3_N_4_, and P-g-C_3_N_4_ nanoparticles synthesized as conductive materials. The diffraction peaks that appeared at (002) and (100) crystal planes of hexagonal phase g-C_3_N_4_ were well matched with JCPDS card No. 01-087-1526. Moreover, these peaks are well compatible with tri-s-triazine-based structural units. It is well known that the different doping atoms into the g-C_3_N_4_ structure lead to changes in morphology, crystal structure, particle size, location of diffraction angles, and intensity of peaks. Figure S3a (Supporting Information) reveals the XRD pattern of O-doped g-C_3_N_4_ nanoparticles, which have two broad diffraction peaks at 2θ = 13.1° and 27.6°. The main peaks in the O-g-C_3_N_4_ nanoparticles are shifted to larger angles than those of graphite nitride carbon (2θ = 12.8° and 27.3°). This is probably due to the high electronegativity of the oxygen atoms relative to the atoms of carbon and nitrogen, resulting in a reduction in the distance between the inner plates^32^. The X-ray diffraction pattern of S-g-C_3_N_4_ nanoparticles is shown in Fig. S3b (Supporting Information). According to this pattern, two diffraction peaks were observed at 2θ = 12.6° and 27.2°. It is clearly visible that the main peaks in the synthesized nanoparticles are transferred to smaller angles than those of graphite nitride carbon. This indicates that the plates’ distance has been increased due to the larger radius of the sulfur atoms^33^. Figure S3c (Supporting Information) shows the XRD pattern of P-g-C_3_N_4_, which has the main peaks at 2θ = 12.5° and 26.9°. The diffraction peaks observed in this pattern are shifted to smaller angles than those of graphite nitride carbon. This shows that the distance between the inner plates has been enhanced owing to the larger radius of the phosphorus atoms^34^. The crystallite sizes of O-g-C_3_N_4_, S-g-C_3_N_4_, and P-g-C_3_N_4_ nanoparticles were calculated about 14.94, 9.35, and 14.50 nm, respectively (Scherrer formula). Figure 2a represents the SEM images of synthesized NiFe_2_O_4_ nanoparticles whose particle size is about 21–29 nm. As can be seen, the severe agglomeration has been occurred between particles, which could own to the intrinsic properties of the magnetic ferrites. The SEM images of MgTiO_3_, ZnTiO_3_, and FeTiO_3_ nanoparticles are shown in Fig. 2b–d, in which their particle sizes are about 18–60, 25–31, and 28–45 nm. As can be seen from the images, the synthesized nanoparticles are observed as the particles agglomerate. This is probably due to the rapid particle growth (the increasing particle size) and reduced particle density at high temperatures. The particle size, chemical composition, and surface morphology of the synthesized O-g-C_3_N_4_, S-g-C_3_N_4_, and P-g-C_3_N_4_ were studied using SEM, TEM, EDX, and the SEM-elemental map analysis, respectively. The SEM, TEM, EDX, and SEM-elemental mapping images of the O-g-C_3_N_4_ nanoparticles are indicated in Fig. 3a,b and S4a,b (Supporting Information), respectively. According to the SEM images, the surface of the synthesized nanoparticles is glabrous with a particle size of 26–50 nm. Meanwhile, the TEM image indicates that the O-g-C_3_N_4_ nanoparticles have nanosheet morphology with ultrathin thickness. The darker area in the TEM picture is due to multiple nanosheets overlapping. As can be observed from the EDX analysis, the prepared sample consists of carbon, nitrogen, and oxygen atoms, which confirms the successful synthesis of O-g-C_3_N_4_. Moreover, the oxygen atoms were well dispersed between carbon and nitrogen atoms, which can be observed from SEM-elemental mapping images (Fig. S4b, Supporting Information). Figure 3c presents the SEM images of the S-g-C_3_N_4_ nanoparticles, which have a particle size of about 36–51 nm. Besides, the TEM image of the S-g-C_3_N_4_ nanoparticles (Fig. 3d) clearly exhibitions that these nanoparticles have nanosheet morphology and thin diameter. According to the EDX images (Fig. S5a, Supporting Information), the synthesized sample contains the carbon, nitrogen, and sulfur atoms that it is evidence of the successful synthesis of S-g-C_3_N_4_ nanoparticles. It is clearly visible from the SEM-elemental mapping images (Fig. S5b, Supporting Information) that the sulfur atoms were localized between carbon and nitrogen atoms. According to the SEM and TEM images in Fig. 3e,f, the P-g-C_3_N_4_ nanoparticles have a nanosheet morphology with some irregular cavities and a particle size of 19–26 nm. The darker parts can be related to the accumulation of several flat layers. As can be seen from the EDX and SEM-elemental mapping images (Fig. S6a,b, Supporting Information), the prepared nanocomposite comprises carbon, nitrogen, and phosphorus atoms, and also the phosphorus atoms were well distributed between the nitrogen and carbon atoms; thereby these results confirm the successful synthesis of P-g-C_3_N_4_ nanoparticles.

### Microwave-absorbent coatings properties

**Figure 4:**
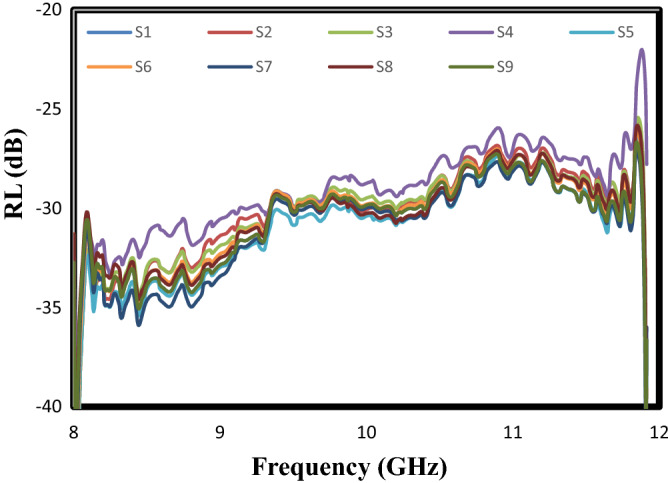
The frequency dependent of the reflection loss (RL) curves of sample prepared based on the Taguchi method; According to Tables 1 and 2, S1: experiment 1, S2: experiment 2, S3: experiment 3, S4: experiment 4, S5: experiment 5, S6: experiment 6, S7: experiment 7, S8: experiment 8, S9: experiment 9.

Reflection loss (RL) is explained as the rate of microwave absorption by nanocomposites. There are three basic ways to calculate this parameter: (1) Transmission-line theory; (2) Radar cross-section measurement; (3) Use of reflection and transition parameters. The transmission-line theory has received much attention from researchers to investigate the microwave absorbing properties by the absorbents. The basis of this method is the design of materials with 100% absorption and zero reflection, so the material impedance must be equal to the free space impedance (Z_in_ = Z_0_). However, it is an ideal situation, in which no thin material has ever been found under such conditions^35^. Reflection loss (RL) based on this method is calculated as follows:

3 $${\text{R}}_{{\text{L}}} \left( {{\text{dB}}} \right) = 20\;\log \Gamma = - 20\log_{10} \left[ {\left| {\frac{{{\text{Z}}_{{{\text{in}}}} - 1}}{{{\text{Z}}_{{{\text{in}}}} + 1}}} \right|} \right]$$

4 $$Z_{in} = Z_{0} \sqrt {\upmu _{r} /\upvarepsilon _{r} } tanh\left[ {\left( { - \frac{j2\pi }{c}} \right)\left( {\frac{{v_{r} }}{{\upvarepsilon _{r} }}} \right)^{1/2} {\text{fd}}} \right]\;{\text{and}}\;Z_{0} = \sqrt {\upmu _{0} /\upvarepsilon _{0} }$$

where Z_in_ and Z_0_ are the material impedance and the free space impedance, respectively; $\upmu _{r}$ is the complex magnetic permeability ($\upmu _{r} =\upmu ^{\prime } - j\upmu ^{\prime \prime }$); $\varepsilon_{r}$ is the complex dielectric permittivity ($\varepsilon_{r} = \varepsilon^{\prime } - j\varepsilon^{\prime \prime }$)^36,37^. Figure 4 presents the reflection loss (RL) curve of the prepared samples based on the L_9_ orthogonal array. It is clearly seen that the whole frequency range of the X-band was covered by the synthesized samples with the reflection loss (RL) values less than − 30 dB. Thereby, the synthesized nanocomposites have absorption of more than 99% and a broadband wide with 1 mm thickness. It displays that the distribution of the prepared nanoparticles in the polyester substrate was well accomplished, so the charge carriers have been localized at the interfaces of these nanofillers^38^. It is worth emphasized that the charge accumulation, in turn, leads to improve microwave absorbing properties and distortion of the electric field^39^. It is essential to notice that the calculated values for the area under the reflection loss (RL) curve (Table 3) were elected as a response in the Taguchi method to obtain the optimal sample. Besides, the Taguchi method uses analysis of variance (ANOVA) to determine the effect and contribution of each of the input parameters in the response. The ANOVA results obtained by Qualitek-4 software are shown in Table 4. As can be seen, the highest percentage of contribution in the response is ascribed to the weight ratio of MTiO_3_/NiFe_2_O_4_: X-doped g-C_3_N_4_ (equal to 44.44%). The contribution percentage of the weight ratio of MTiO_3_:NiFe_2_O_4_, the type of dielectric material, and the type of dopant in g-C_3_N_4_, is 35.69%, 14.82%, and 5.05%, respectively. The contribution percentage of error in this experimental design is zero, due to the completeness of the orthogonal array (L_9_) for the selected factors and their levels.

**Table 3:** The calculated values for the area under of the reflection loss (RL) curves by Qualitek-4 software.

Test	Area under the curves (GHz dB)
1	122.31
2	120.63
3	120.70
4	115.90
5	124.00
6	121.39
7	124.02
8	121.33
9	122.31

**Table 4:** Analysis of variance (ANOVA).

Parameter	DOF (f)	Sum of square (Sʹ)	Variance (v)	Percent (%)
1	2	6.90	3.45	14.82
2	2	2.35	1.18	5.05
3	2	16.62	8.31	35.69
4	2	20.69	10.34	44.44
Other/error	0	0.00		0.00
Total	8			100

**Figure 5:**
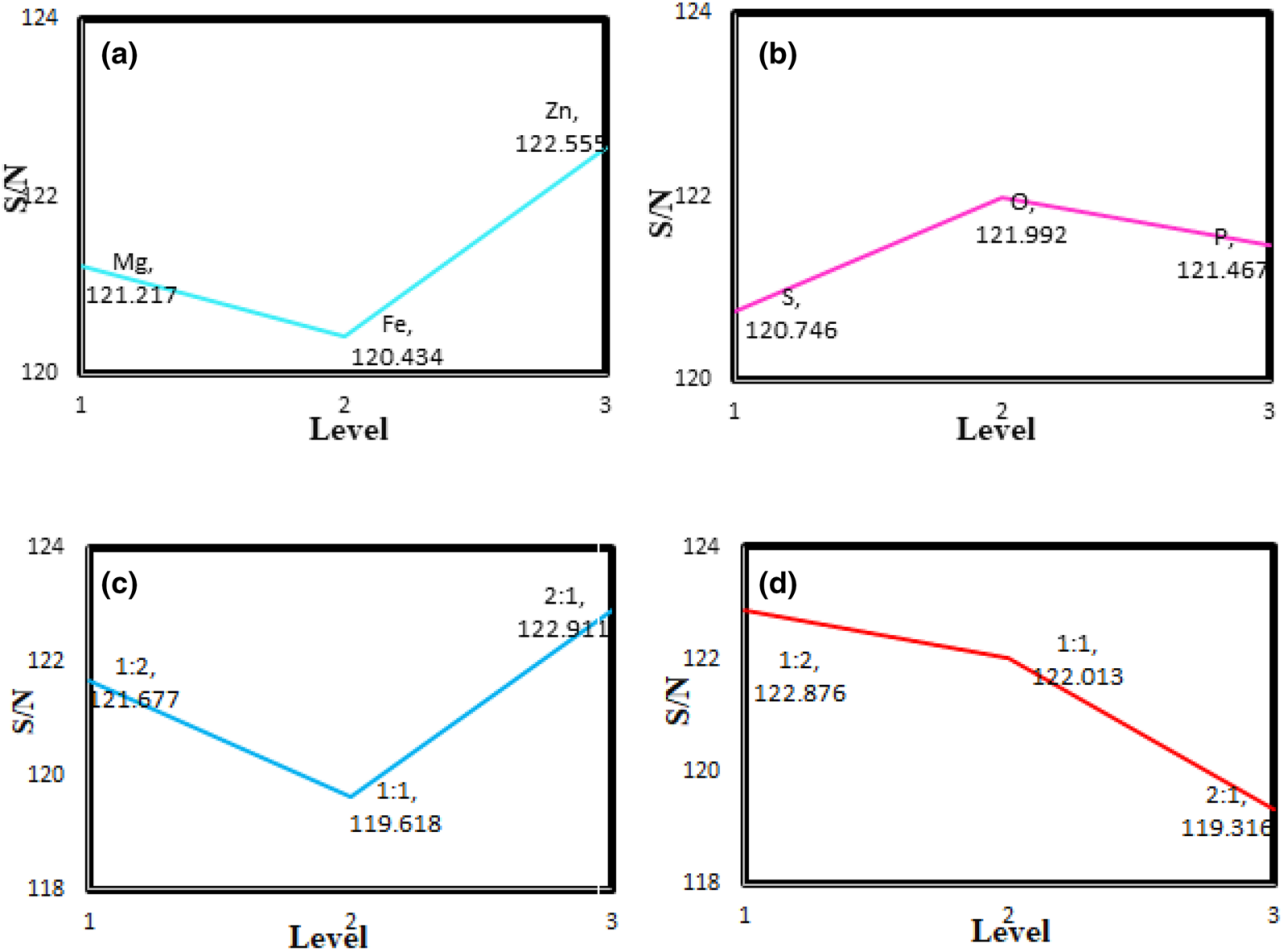
Effect of the levels for (**a**) the type of dielectric material; (**b**) the type of dopant in g-C_3_N_4_; (**c**) the weight ratio of MTiO_3_:NiFe_2_O_4_; (**d**) the total weight ratio of MTiO_3_/NiFe_2_O_4_: X-doped g-C_3_N_4_.

The effect of uncontrollable factors (noise) on the system response should be minimized to determine the optimal conditions in the Taguchi method. Figure 5a presents the influence levels for the type of dielectric material on the S/N ratio. According to this figure, the third level (i.e., ZnTiO_3_) has the most significate effect on the S/N ratio and reduces the reflection from the absorber surface. Figure 5b displays the S/N ratio results versus the levels of the type of dopant in g-C_3_N_4_. Based on the results obtained, oxygen as a dopant in g-C_3_N_4_ has the highest S/N ratio. As a result, it plays a significant role in improving the performance of the adsorbents. Figure 5c indicates the influence of the weight ratio of MTiO_3_:NiFe_2_O_4_ on the performance of microwave absorbents in the X-band. As can be observed, the highest S/N ratio is related to the 2:1 ratio of the dielectric material to the magnetic material. It shows that a high amount of dielectric filler compared to magnetic material leads to an increase in the number of the polarization domain. Figure 5d presents the impact of the total weight ratio of the magnetic and dielectric material to the conductive material on the S/N ratio, in which the 1:2 ratio has the most impact on the absorbent performance.

### Design of optimal nanocomposite

**Figure 6:**
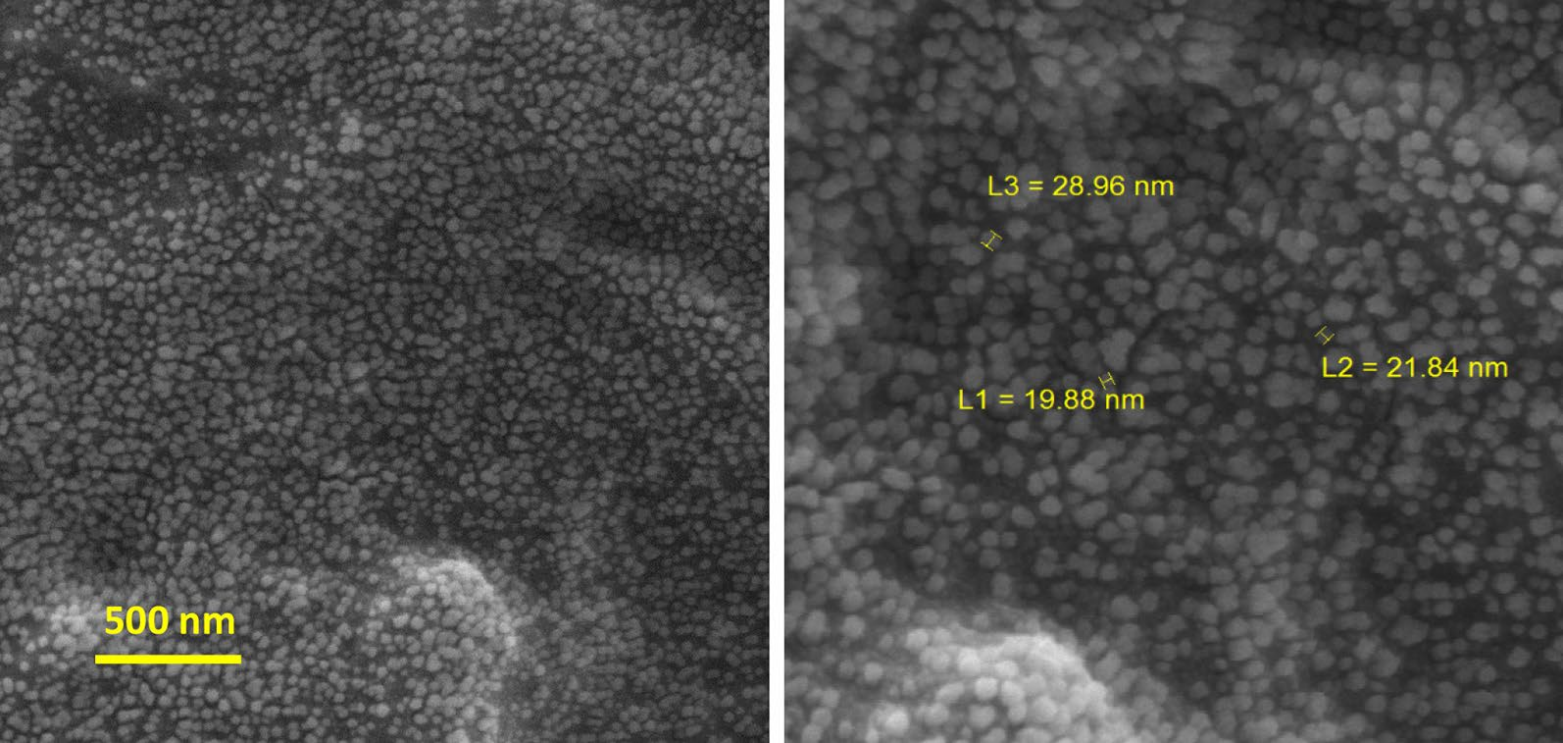
SEM images of the absorber prepared under the optimal conditions.

**Figure 7:**
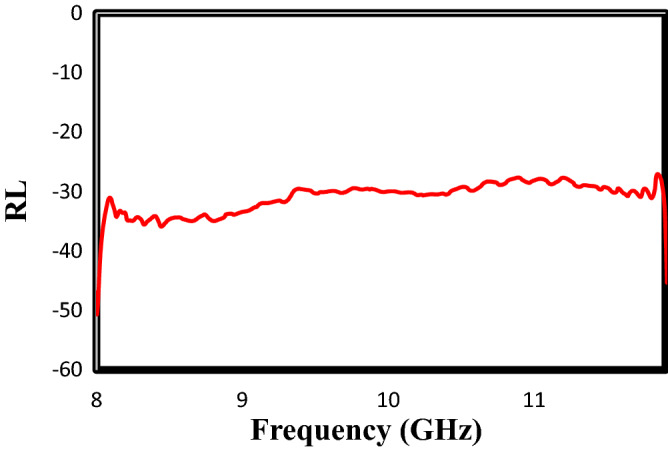
The frequency dependent of reflection loss (RL) curves for the absorber prepared under the optimal conditions at X-band.

In the present work, the Taguchi experimental design was performed to optimize the nanocomposites’s formulation according to the maximum value of the area under the RL curve at X-band. Based on the results found from the ANOVA table, the optimum sample includes ZnTiO_3_, O-g-C_3_N_4_, NiFe_2_O_4_, the 2:1 weight ratio for MTiO_3_:NiFe_2_O_4_, and the 1:2 weight ratio for MTiO_3_/NiFe_2_O_4_: X-doped g-C_3_N_4_ (the most significate parameter). Figure 6 exhibits the surface morphology of the optimum sample, in which the nanofillers with particle sizes about 19–29 nm were well distributed in the polyester substrate. Figure 7 shows the frequency dependence of the RL curve for the optimum sample at 1 mm thickness. As can be seen, this nanocomposite has a reflectance loss (RL) of less than − 30 dB, equivalent to 99.9% absorption, and broad bandwidth. Moreover, the area under the RL curve for the optimal nanocomposite was calculated 125.38 dB, which confirms the value predicted by the experimental design software (126.19 dB) with a slight difference. More importantly, Table 5 displays the microwave absorption properties of some synthesized absorbers over several recent years. Strong absorption combined with wide bandwidth and a thin thickness made the optimal nanocomposite a highly competitive absorbent. Therefore, the ZnTiO_3_, O-g-C_3_N_4_, NiFe_2_O_4_, and polyester nanocomposite in this work was promising as a microwave absorbing material.

**Table 5:** The microwave absorption performance of some reported absorbents over several recent years.

Nanocomposite	Optimal RL value (dB)	Frequency of Maximum RL (GHz)	Bandwidth (GHz) RL < − 10 dB	Thickness (mm)	Frequency range (GHz)	References
CoFe_2_O_4_ nanoparticles-decorated Ti_3_C_2_ MXene	− 30.9	13.9	8.5	1.5	8.3–16.8	^13^
Copper-cobalt–nickel ferrite/graphene oxide/polyaniline	− 33.0	10.8	–	2.0	2–18	^7^
MnFe_9n+3_O_15n+4_(0 ≤ n ≤ 1) (M = Ba, Sr)/CaCu_3_Ti_4_O_12_/phosphorus-doped g-C_3_N_4_	− 30.0	X-band	4.0	1.0	8–12	^16^
(Ba_0.5_Sr_0.5_Fe_12_O_19_)_0.6_/(NiFe_2_O_4_)_0.4_	− 38.7	8.26	–	2.5	8–12	^14^
g-C_3_N_4_ and polyester	− 10.0	X-band	4.0	2.0	8–12	^6^
SrTiO_3_/SrFe_12_O_19_/ polyaniline	− 15.0	9.2	1.6	1.0	8–12	^40^
La doped barium titanate	− 41.0	9.8	1.7	2.0	8–12	^41^
ZnTiO_3_, O-g-C_3_N_4_, NiFe_2_O_4_, and polyester	− 30.0	X-band	4.0	1	8–12	This work

**Figure 8:**
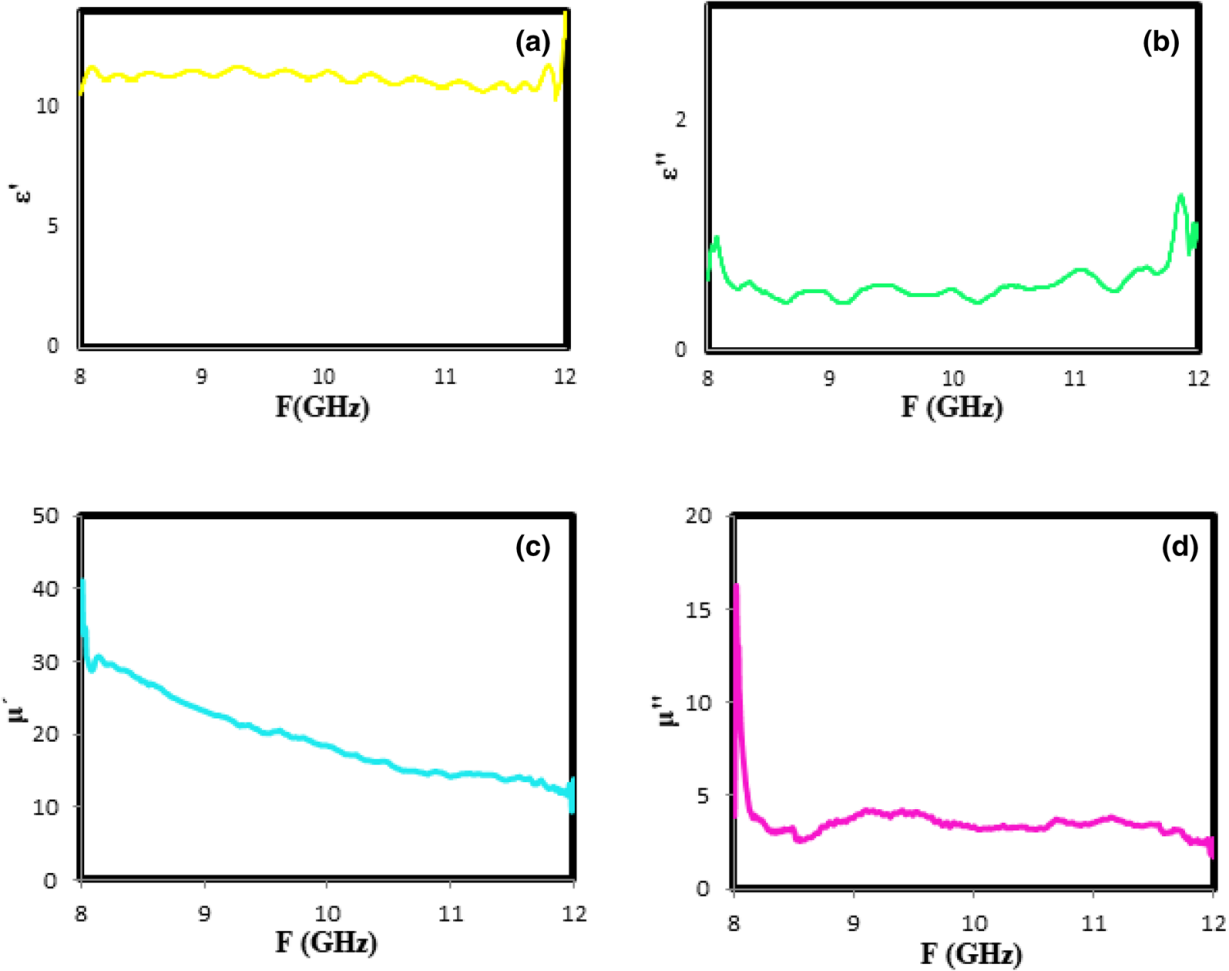
The relative complex permeability (**a**) real part ($\mu^{\prime }$) and (**b**) imaginary part ($\mu^{\prime \prime }$); the relative complex permittivity (**c**) real part ($\varepsilon^{\prime }$) and (**d**) imaginary part ($\varepsilon^{\prime \prime }$) at X-band for optimum sample.

The $\upmu _{r}$,$\varepsilon_{r}$, and Γ were calculated using the direct method of the Nicholson–Ross–Weir (NRW) algorithm and the scattering parameters. According to NRW, The reflection coefficient (Γ) can be calculated as follows:

5 $$\Gamma = \frac{{s_{11}^{2} - s_{21}^{2} + 1}}{{2s_{11} }} \pm \sqrt {\left( {\frac{{s_{11}^{2} - s_{21}^{2} + 1}}{{2s_{11} }}} \right)^{2} - 1} ,\;\left| \Gamma \right| < 1$$

where S_11_ and S_21_ are the reflected and transmitted signals obtained by the vector network analyzer, respectively^42^. The plots of the complex electric permittivity ($\varepsilon_{r}$) and the complex magnetic permeability ($\upmu _{r}$) versus the frequency can be used to determine the microwave absorption mechanism by the prepared absorbent in the optimal conditions. For this purpose, the real parts of the complex expressions (i.e., $\upmu ^{\prime }$ and $\varepsilon^{\prime }$) are attributed to the energy storage in the absorbers, and the imaginary parts (i.e., $\varepsilon^{\prime \prime }$ and $\mu^{\prime \prime }$) are related to the energy loss and its conversion to heat by absorbers. Figure 8a,b displays the variations of the real ($\varepsilon^{\prime }$) and imaginary ($\varepsilon^{\prime \prime }$) parts of the complex electric permittivity for the optimal sample in the X-band frequency range. According to these figures, the $\varepsilon^{\prime }$ and $\varepsilon^{\prime \prime }$ are independent of the frequency variation, which shows the dielectric constant of ZnTiO_3_ as the dielectric material is free from the used frequency range. Figure 8c,d shows the frequency dependence of $\upmu ^{\prime }$ and $\mu^{\prime \prime }$ for the prepared sample under the optimum conditions. As shown in Fig. 8c, the $\upmu ^{\prime }$ values decrease with a gentle slope from 35 to 10 as the frequency increases. It is ascribed to the high conductivity of O-g-C_3_N_4_ and thereby increasing the interfacial and dipolar polarization. According to Fig. 8d, no change in the $\mu^{\prime \prime }$ values is observed with the changing frequency from 8 to 12 GHz.

**Figure 9:**
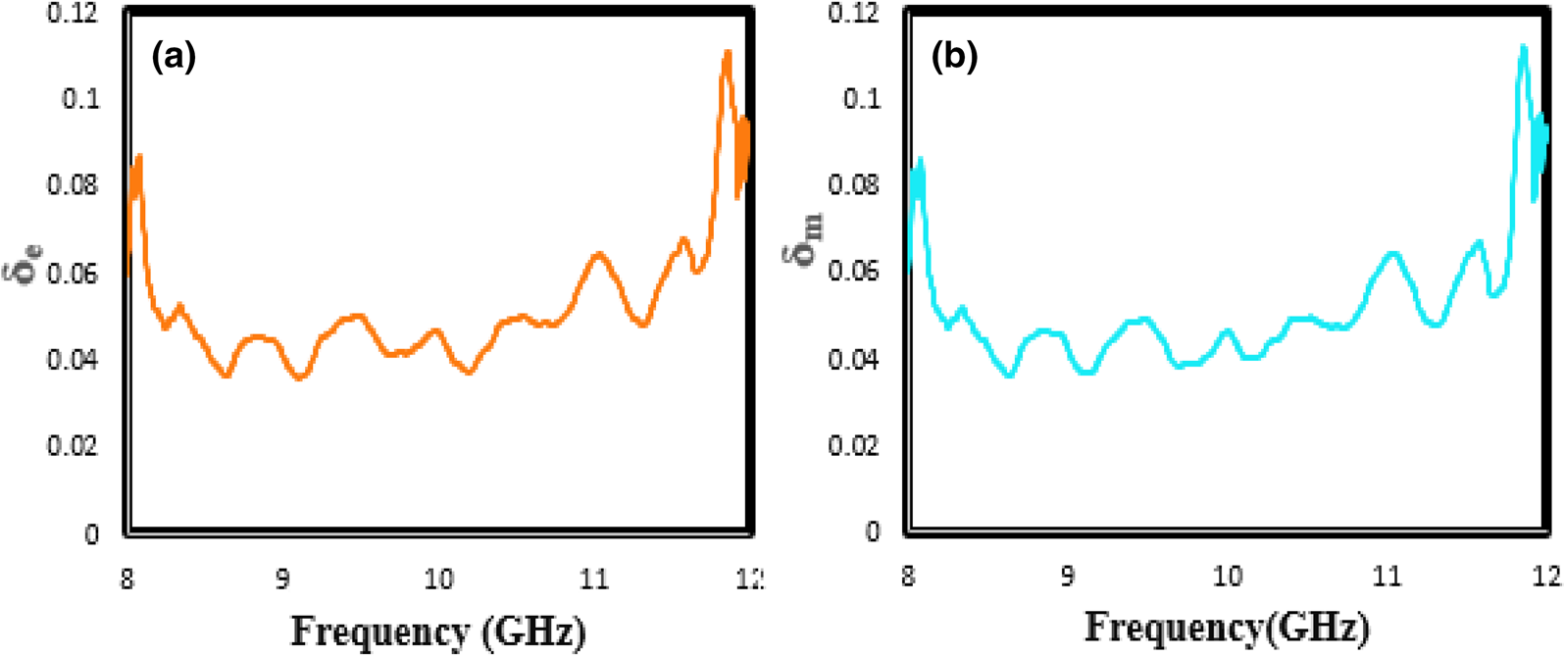
The tangent curves of (**a**) the electrical permeability (δ_e_) (**b**) the magnetic permittivity (δ_m_) versus frequency indicated to optimal sample.

Dielectric loss tangent (tan (δ_e_) = $\varepsilon^{\prime \prime }$/$\varepsilon^{\prime }$) and magnetic loss tangent (tan (δ_m_) = $\mu^{\prime \prime }$/$\mu^{\prime }$) can be used to better investigate the microwave absorption characteristics in optimum nanocomposite. In other words, as the magnetic and dielectric loss tangents increases, the microwave absorber efficiency increases. According to Fig. 9a,b, both tan (δ_m_) and tan (δ_e_) for the optimal sample are dominated in the range of 0.06–0.1, which should benefit to achieve good impedance matching.

**Figure 10:**
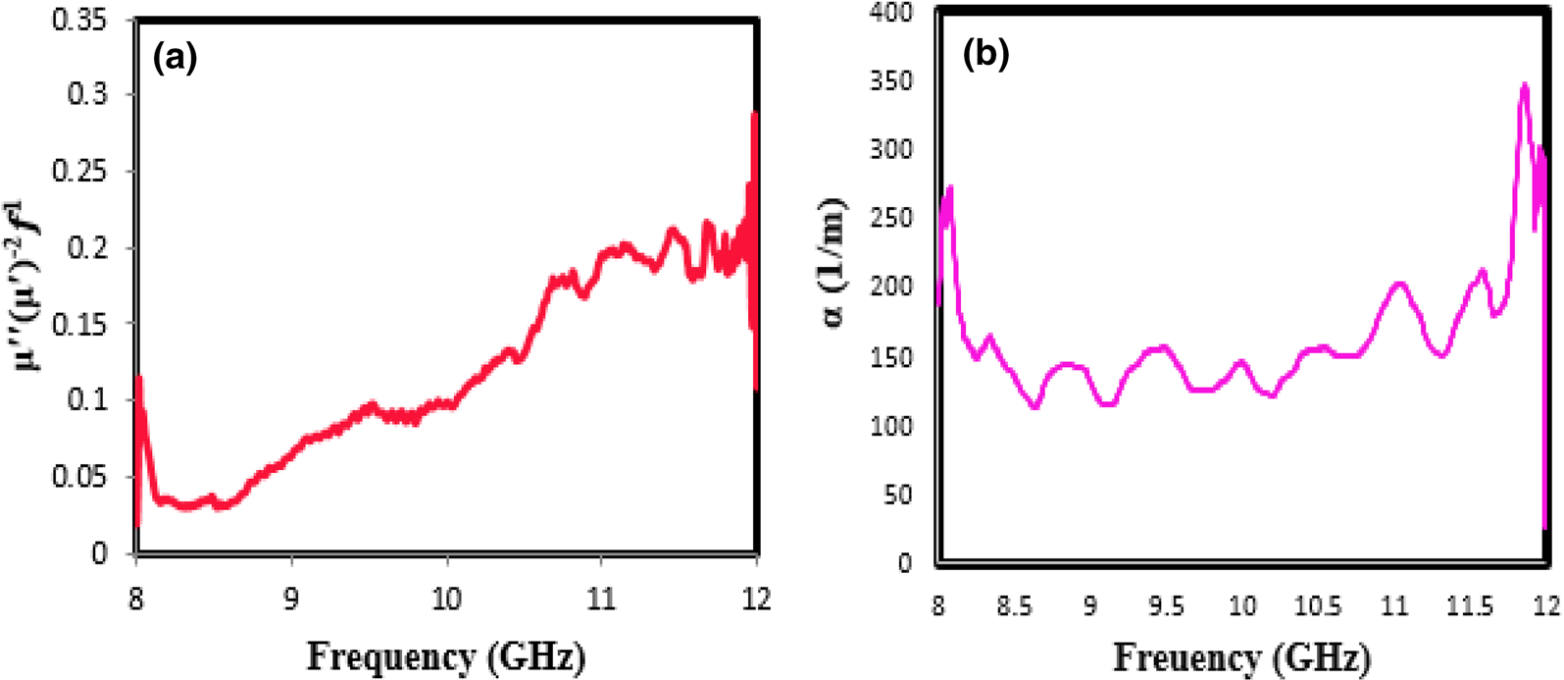
The values of (**a**) $\mu^{\prime\prime}$($\mu^{\prime}$)^−2^*f*^1^; (**b**) attenuation constant (α) versus frequency for optimal sample.

Factors influencing magnetic loss in a magnetic material include eddy current effect, domain-wall resonance, hysteresis loss, and natural resonance. The hysteresis loss is due to the irreversible magnet, which occurs in a highly functional field and can be omitted at a high frequency and reduced applied field. The domain-wall resonance from multi-domain materials usually appears in the range of 100–100 MHz. Besides, if the $\mu^{\prime \prime }$($\mu^{\prime }$)^−2^*f*^1^ value remains constant with frequency-changing, the magnetic loss is due to the eddy current loss^43^. The values of $\mu^{\prime \prime }$($\mu^{\prime }$)^−2^*f*^1^ vary significantly with increasing frequency, as shown in Fig. 10a. As a result, the magnetic loss for the prepared sample under the optimal condition is due to natural resonance.

Another important factor to better understand the behavior of microwave absorbents is the use of attenuation constant ($\alpha$), which can be expressed as the following equation:

6 $$\alpha = \frac{\sqrt 2 \pi f}{c} \times \sqrt {\left( {\mu^{\prime \prime } \varepsilon^{\prime \prime } - \mu^{\prime } \varepsilon^{\prime } } \right) + \sqrt {\left( {\mu^{\prime \prime } \varepsilon^{\prime \prime } - \mu^{\prime } \varepsilon^{\prime } } \right)^{2} + \left( {\varepsilon^{\prime } \mu^{\prime \prime } + \varepsilon^{\prime \prime } \mu^{\prime } } \right)^{2} } }$$

According to Eq. (6), the attenuation constant depends on the two parameters of dielectric loss and magnetic loss; increasing the last two parameters leads to further attenuation of the microwave waves or increasing the attenuation constant^44,45^. Figure 10b shows the plot of attenuation constant ($\alpha$) versus frequency range from 8 to 12 GHz for the optimum sample. As shown in this figure, the synthesized sample has further $\alpha$ (more than 200 dB/m) at the frequency range (X-band), which confirms the severe attenuation of the microwave in the absorber.

## Conclusions

In summary, a novel microwave-absorber polyester coating containing the nanofillers of NiFe_2_O_4_, X-doped g-C_3_N_4_ (M = S, P, and O), and MTiO_3_ (M = Fe, Mg, and Zn) was successfully designed. Based on the results obtained from the Taguchi method, the optimum conditions for the synthesis of a great microwave absorbent included: ZnTiO_3_, O-g-C_3_N_4_, NiFe_2_O_4_, the 2:1 weight ratio for MTiO_3_:NiFe_2_O_4_, and the 1:2 weight ratio for MTiO_3_/NiFe_2_O_4_: X-doped g-C_3_N_4_. The optimal sample has reflection loss (RL) values less than − 30 dB at a high-frequency range (8–12 GHz) with a thickness of 1 mm. It was found that the combination of the conductor, magnetic, and dielectric materials leads to good impedance matching and increasing dipolar polarization. Moreover, the high values of magnetic loss tangent, dielectric loss tangent, and attenuation constant demonstrated the ability of the prepared coatings for the absorption and the conversion of electromagnetic energy into heat at X-band. Thus, the optimal nanocomposite proposed in this study can be used as a promising absorber with low density and thickness.

## Supplementary Information

Supplementary Figures.
