# Supplementary material for: Improvement of microwave absorption properties of polyester coatings using NiFe2O4, X-doped g-C3N4 (X = S, P, and O), and MTiO3 (M = Fe, Mg, and Zn) nanofillers
Source: Sci Rep. 2021 Sep 29;11:19339. doi: 10.1038/s41598-021-98666-6 (PMC8481508; doi:10.1038/s41598-021-98666-6)
Supplement: Supplementary file 1 — Supplementary Figures. [file 41598_2021_98666_MOESM1_ESM.docx]

**Supplementary Information**

**Improvement of microwave absorption properties of polyester coatings using NiFe_2_O_4_, X-doped g-C_3_N_4_ (X= S, P, and O), and MTiO_3_ (M= Fe, Mg, and Zn) nanofillers**

Somayeh Solgi^1^, Mir Saeed Seyed Dorraji*^1^, Seyyedeh Fatemeh Hosseini^1^, Mohammad Hossein Rasoulifard^1^, Ismael Hajimiri^1^, Alireza Amani-Ghadim^2^

1. Applied Chemistry Research Laboratory, Department of Chemistry, Faculty of Science, University of Zanjan, Zanjan, Iran.
2. Department of Chemistry, Faculty of Science, Azarbaijan Shahid Madani University, P.O. box 83714-161, Tabriz, Iran.

Corresponding authors:* Email: [dorraji@znu.ac.ir](mailto:dorraji@znu.ac.ir)

Tel: 024 3305 2591

Fax: 024 3305 2477

**Fig. S1** XRD pattern of NiFe_2_O_4_ nanoparticles


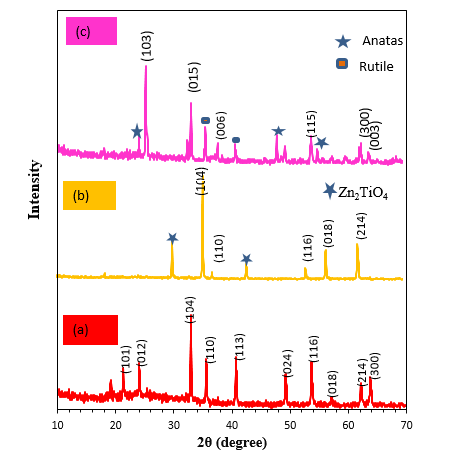


**Fig. S2** XRD pattern of (a) MgTiO_3_; (b) ZnTiO_3_; (c) FeTiO_3_ nanoparticles


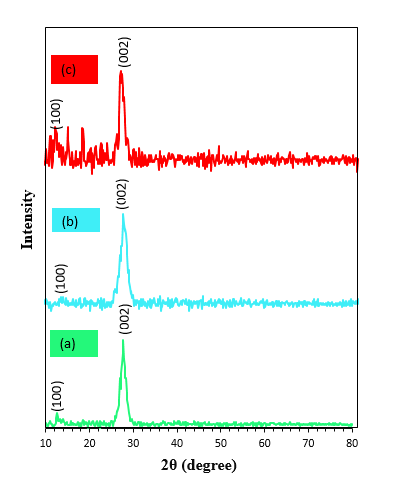


**Fig. S3** XRD pattern of (a) O-g-C_3_N_4_; (b) S-g-C_3_N_4_; (c) P-g-C_3_N_4_ nanoparticles


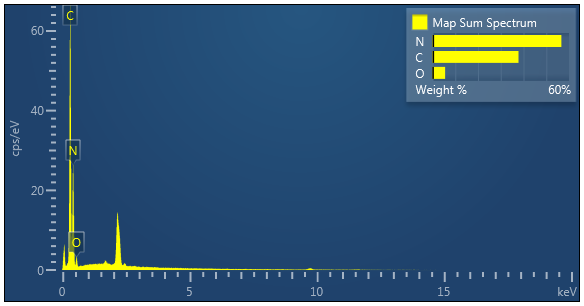


(a)


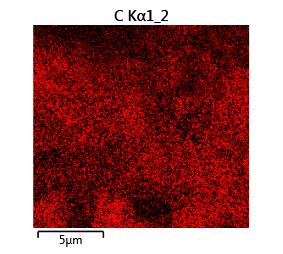

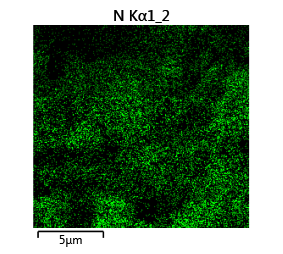

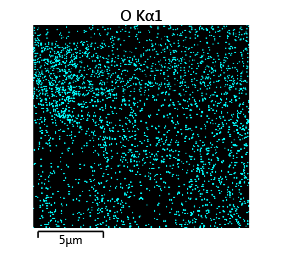


(b)

**Fig. S4** (a) EDX spectrum; (b) SEM-elemental maps of O-g-C_3_N_4_ nanoparticles


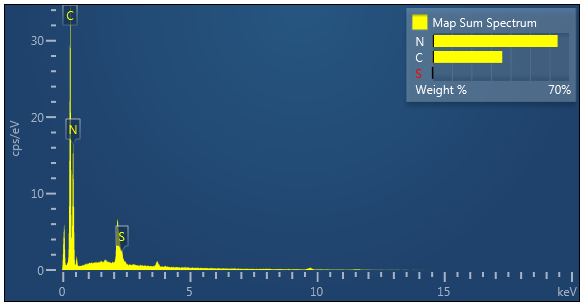


(a)


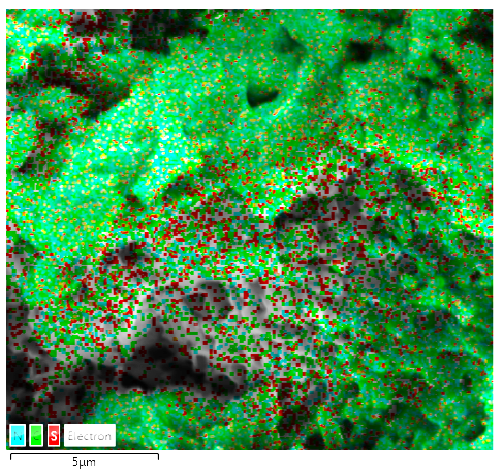

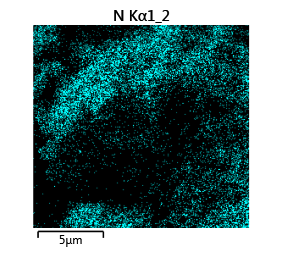

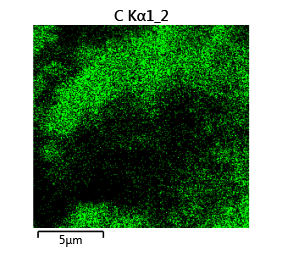

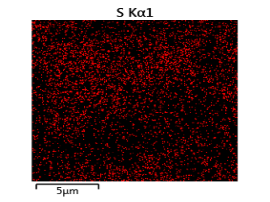


(b)

**Fig. S5** (a) EDX spectrum; (b) SEM-elemental maps of S-g-C_3_N_4_ nanoparticles


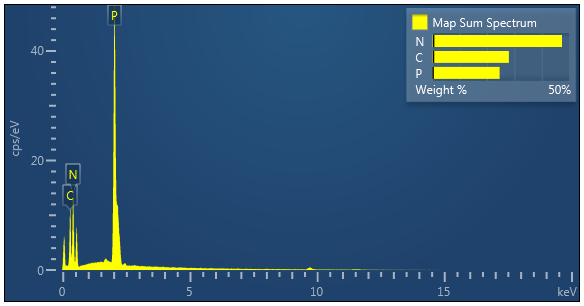


(a)


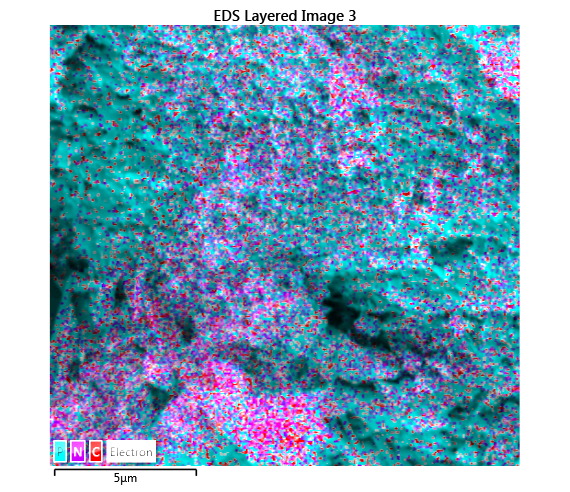

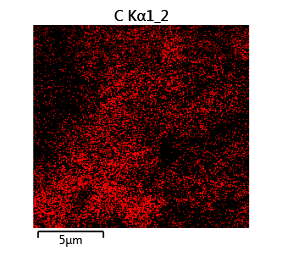

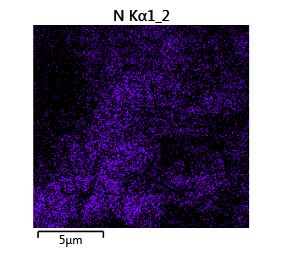

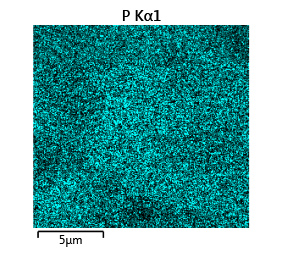


(b)

**Fig. S6** (a) EDX spectrum; (b) SEM-elemental maps of P-g-C_3_N_4_ nanoparticles
